# Novel Anti-Campylobacter Compounds Identified Using High Throughput Screening of a Pre-selected Enriched Small Molecules Library

**DOI:** 10.3389/fmicb.2016.00405

**Published:** 2016-04-06

**Authors:** Anand Kumar, Mary Drozd, Ruby Pina-Mimbela, Xiulan Xu, Yosra A. Helmy, Janet Antwi, James R. Fuchs, Corey Nislow, Jillian Templeton, Patrick J. Blackall, Gireesh Rajashekara

**Affiliations:** ^1^Food Animal Health Research Program, Department of Veterinary Preventive Medicine, The Ohio State UniversityWooster, OH, USA; ^2^Poultry CRC, University of New EnglandArmidale, NSW, Australia; ^3^Department of Plant Pathology, Ohio Agricultural Research and Development Center, The Ohio State UniversityWooster, OH, USA; ^4^College of Pharmacy, The Ohio State UniversityColumbus, OH, USA; ^5^Pharmaceutical Sciences, The University of British ColumbiaVancouver, BC, Canada; ^6^Department of Agriculture and Fisheries, EcoSciences PrecinctDutton Park, QLD, Australia; ^7^Queensland Alliance for Agriculture and Food Innovation, The University of Queensland, EcoSciences PrecinctDutton Park, QLD, Australia

**Keywords:** *Campylobacter*, small molecules, enriched small molecules library, high throughput screening, food safety

## Abstract

*Campylobacter* is a leading cause of foodborne bacterial gastroenteritis worldwide and infections can be fatal. The emergence of antibiotic-resistant *Campylobacter* spp. necessitates the development of new antimicrobials. We identified novel anti-*Campylobacter* small molecule inhibitors using a high throughput growth inhibition assay. To expedite screening, we made use of a “bioactive” library of 4182 compounds that we have previously shown to be active against diverse microbes. Screening for growth inhibition of *Campylobacter jejuni*, identified 781 compounds that were either bactericidal or bacteriostatic at a concentration of 200 μM. Seventy nine of the bactericidal compounds were prioritized for secondary screening based on their physico-chemical properties. Based on the minimum inhibitory concentration against a diverse range of *C. jejuni* and a lack of effect on gut microbes, we selected 12 compounds. No resistance was observed to any of these 12 lead compounds when *C. jejuni* was cultured with lethal or sub-lethal concentrations suggesting that *C. jejuni* is less likely to develop resistance to these compounds. Top 12 compounds also possessed low cytotoxicity to human intestinal epithelial cells (Caco-2 cells) and no hemolytic activity against sheep red blood cells. Next, these 12 compounds were evaluated for ability to clear *C. jejuni in vitro*. A total of 10 compounds had an anti-*C. jejuni* effect in Caco-2 cells with some effective even at 25 μM concentrations. These novel 12 compounds belong to five established antimicrobial chemical classes; piperazines, aryl amines, piperidines, sulfonamide, and pyridazinone. Exploitation of analogs of these chemical classes may provide *Campylobacter* specific drugs that can be applied in both human and animal medicine.

## Introduction

Illness associated with *Campylobacter*, termed “campylobacteriosis,” is one of the most common forms of foodborne gastroenteritis in developed countries (Blaser and Engberg, 2008) and is a greater burden in developing countries where *Campylobacter* spp. associated diarrhea and bacteremia cases are seen in HIV/AIDS patients (Coker et al., 2002; Scott, 2003). Further, *Campylobacter* species have recently been associated with the Inflammatory Bowel Diseases such as Crohn's and ulcerative colitis (Kaakoush et al., 2014a,b).

*Campylobacter jejuni* is a zoonotic pathogen and accounts for nearly 90% of the total human *Campylobacter* infections, while *C. coli* infections constitute 6% of cases (Friedman et al., 2004). The illnesses are usually sporadic, although rare outbreaks have been reported. Ingestion of raw or undercooked poultry is a major source of human campylobacteriosis; however, disease can be contracted by drinking unpasteurized milk or contaminated water (Engberg et al., 1998; Allos, 2001; Blaser and Engberg, 2008). Clinical signs of infection are typical of self-limiting foodborne gastroenteritis with fever, vomiting, headache, diarrhea, and abdominal pain. A small percentage of cases can lead to fatal complications like reactive arthritis and Guillain-Barr'e syndrome (Tam et al., 2006; Pope et al., 2007).

Antimicrobial therapy is warranted in severe disease manifestations and in immune-compromised individuals. The most commonly used antibiotics are macrolides (e.g., erythromycin) and fluoroquinolones (e.g., ciprofloxacin) with tetracycline used as an alternative choice (Moore et al., 2006; Blaser and Engberg, 2008). Intravenous use of aminoglycosides is also recommended in serious cases of *Campylobacter* infections, such as bacteremia and systemic infections (Saenz et al., 2000; Aarestrup and Engberg, 2001). As the use of antibiotics for therapy and prophylaxis increases in both human and animal medicine, increasing numbers of *Campylobacter* isolates have developed resistance to fluoroquinolones, macrolides, aminoglycosides, and beta lactam antibiotics (Aarestrup and Engberg, 2001; Wieczorek and Osek, 2013). Use of fluoroquinolones in poultry production coincides with the emergence of ciprofloxacin resistant *Campylobacter* in humans (Moore et al., 2006). Recent studies have also suggested that use of macrolides and fluoroquinolone classes of antibiotics in food animals may increase the risk of emergence and transmission of antibiotic resistant *C. jejuni* in humans (Kashoma et al., 2015; Klein-Jobstl et al., 2016). Resistance corresponds to active site mutations in DNA gyrase subunit A as well as mutations in the *cmeABC* multidrug efflux pump (Wieczorek and Osek, 2013; Kovac et al., 2015). In addition to spontaneous mutations, *Campylobacter* can acquire resistance by horizontal gene transfer via natural transformation, transduction or conjugation (Perez-Boto et al., 2014).

As campylobacteriosis is projected to remain one of the top ten bacterial conditions globally (Coker et al., 2002), and several antibiotics are no longer effective in treatment of campylobacteriosis (Wieczorek and Osek, 2013), a new generation of effective antimicrobials is critically needed. High-throughput, robust, cost-effective, phenotypic cell-based screening is one such amenable approach to expedite anti-campylobacter molecules discovery. The value of using focused bioactive-enriched libraries compared to large, naïve library screens has been shown in earlier studies (Inglese and Hasson, 2011; Wallace et al., 2011). In the current study, we have screened a pre-selected bioactive small molecule library of 4182 compounds against highly pathogenic *C. jejuni* 81-176 strain. Seventy nine candidate compounds were further selected for secondary screening to evaluate; (i) spectrum of activity on diverse *C. jejuni* strains, (ii) activity against commensal/probiotic bacteria, (iii) Minimum Inhibitory Concentrations (MIC), (iv) Minimum Bactericidal Concentrations (MBC), (v) ability of *C. jejuni* to develop resistance, (vi) cytotoxicity and hemolytic activity, and (vii) clearance of *C. jejuni in vitro*. Based on these studies we report potential 12 lead compounds which provide chemical scaffolds for *Campylobacter*-specific antimicrobial development in the future.

## Materials and methods

### Small molecules library and bacterial strains

In prior work, a library of 81,320 small, drug-like molecules was obtained from Chembridge (San Diego, CA) and screened for growth inhibition against diverse prokaryotes and eukaryotic spp. including human lung cancer cell line to yield a enriched library with 4182 bioactive compounds (Wallace et al., 2011; Xu et al., 2015). This library has been made available for purchase as a pre-selected compound set through Chembridge, Inc. All these data is available at Wallace et al. (2011). We have also provided a prioritized list of compounds generated by using our model in the supplementary website: (http://chemogenomics.med.utoronto.ca/supplemental/bioactive/index.php).

The bioactive library of 4182 compounds identified in this prior study was purchased from ChemBridge and dissolved in 100 μL dimethyl sulfoxide (DMSO) at a stock concentration of 20 mM in 96-well plate. Four copies of the library (25 μL of compounds/plate) were prepared and stored at −80°C to maximize sample longevity and potency and minimize the number of freeze/thaw cycles. Bacterial strains, culture conditions and media used in this study are listed in Table S1.

### Primary screen

A primary screen was conducted using the highly invasive *C. jejuni* strain 81–176 originally isolated from a diarrheic patient (Korlath et al., 1985). The *C. jejuni* strains were routinely grown on Mueller-Hinton (MH; Becton Dickinson and Company, MD) agar under microaerobic conditions (85% N_2_, 10% CO_2_, and 5% O_2_) in a DG250 Microaerophilic Workstation (Microbiology International, MD) for 18 h, washed with MH broth and adjusted to a final OD_600_ of 0.1 in MH broth. One hundred micro liter of culture was transferred to 96-well plates and 1 μL (200 μM/μL) of library compounds and controls were added using a pin tool (Wallace et al., 2011). Growth controls including DMSO (1% final concentration), kanamycin (50 μg), and chloramphenicol (20 μg) were added to duplicate wells along with 100 μL un-inoculated MH broth as sterility control. The final concentration was 200 μM for each small molecule. Prior to growth, an aluminum foil was applied (to avoid drug degradation) and plates were then incubated at 42°C, under microaerobic conditions for 24 h. The end-point OD_600_ was measured in a Sunrise TM Tecan plate reader (Tecan Group Ltd. San Jose, CA). We initially tried to exploit the automatic kinetic OD reader option of the Sunrise TM Tecan system but were un-successful. Maintenance of the microaerophilic growth conditions required by *C. jejuni* was difficult in the Tecan reader which is ideally suited for aerobic bacteria. Therefore, we used an end point assay. The quality of high-throughput screen (HTS) was evaluated by calculating the Z′-factor as described previously (Zhang et al., 1999). Z′-factor is defined as 1−((3σ_p_ + 3σ_n_)/|μ_p_ − μ_n_|)) where “σ_p_” and “σ_n_” are the standard deviations of the positive (culture+ DMSO) and negative (culture+antibiotics) controls, respectively and “μ_p_” and “μ_n_” are the means of the positive and negative controls, respectively. A Z′-factor >0.5 indicates a robust assay (Zhang et al., 1999).

The percentage of *C. jejuni* growth inhibition was calculated as mentioned previously (De La Fuente et al., 2006) using the following formula: percentage of inhibition = 100 × (OD negative control–OD test compound)/(OD negative control–OD positive control). Compounds that inhibited *Campylobacter* growth ≥ 99.0% were selected as primary hits. The culture in wells with ≥ 99% growth inhibition were streaked onto fresh MH agar, as were the sterility, antibiotic and no compound control wells. Bacterial growth was measured on the plate after 48 h at 42°C. Based on the recovery of *C. jejuni* on MH agar plates, the compound was classified as either “bacteriostatic” or “bactericidal.”

### Prioritization of bactericidal compounds for secondary screens

A structural analysis of the primary screen data set for 478 bactericidal compounds was conducted. The structures of the compounds in the data set were obtained from online vendors via Supplier ID search. The structural descriptor strings (SMILES) were subsequently converted into ChemDraw (PerkinElmer Inc, Massachusetts, USA) structures using ChemDraw for Excel. The compounds were exported to ChemDraw as a SD file using ChemFinder. The ChemFinder analysis resulted in the rapid identification of compounds containing the same structural motifs. The ChemDraw files of the bactericidal hits were manually sorted into structural groups to establish preliminary structure-activity relationships (SAR). Finally, hits were prioritized for secondary screens based on their adherence to Lipinski's rule of 5 (Lipinski et al., 2001).

### Secondary screens

Seventy nine selected hits were resynthesized from ChemBridge and were dissolved in DMSO in a 96-well plate at the final concentration of 20 mM. The purpose of secondary screen included: (1) testing hits for broad *Campylobacter* spectrum effect, (2) testing the effect of selected hits on Gram positive and Gram negative probiotic/commensal bacteria, (3) determining the minimum inhibitory concentration (MIC), minimum bactericidal concentration (MBC) of the selected hits against *C. jejuni* 81–176, (4) investigating whether *C. jejuni* develop resistance to these compounds, (5) evaluating selected compounds cytotoxicity to human intestinal epithelial cells and sheep RBCs, and (6) assessing effect on intracellular survival of *C. jejuni* 81–176.

### *Campylobacter* spectrum effect

We selected 23 diverse *C. jejuni* strains of different known genotypes to screen for broad *Campylobacter* spectrum effect. The selected 23 *C. jejuni* isolates were further classified into 5 groups (4 or 5 strains/group) based on their single nucleotide polymorphism type as previously established (Table S2; Merchant-Patel et al., 2008). The rationale behind grouping *C. jejuni* isolates was to prepare a pooled culture of strains to screen against all 79 hit compounds, maximizing the throughput and reducing compound consumption. Briefly, the strains (4 to 5 strains; Table S2) were mixed in equal proportion to an OD_600_ of 0.1. One hundred micro liter of the pooled culture was transferred to each well of a 96 well plate in the presence of 200 μM of small molecule and growth inhibition was assessed as above.

### Screening against probiotic/commensal bacteria

The selected compounds were tested for activity against Gram positive (*Bifidobacterium adolescentis, Bifidobacterium longum, Bifidobacterium lactis, Enterococcus faecalis, Lactobacillus brevis, Lactobacillus rhamnosus*) and Gram negative (*Escherichia coli* Nissle 1917) bacteria that were either commonly used probiotics or commensal bacteria. Briefly, the OD adjusted (OD_600_ of 0.05) cultures were treated with 1 μL of compounds (200 μM) and incubated at specific culture conditions (Table S1). At the end point, wells showing significantly decreased OD were further assessed for bactericidal and bacteriostatic effect as described above.

### Minimum inhibitory concentration (MIC)

The dose-dependent effect of 35 compounds against the highly pathogenic *C. jejuni* 81–176 strain was tested. Compounds were two-fold serially diluted (200 to 12.5 μM). One hundred micro liter of the OD_600_ 0.1 adjusted *C. jejuni* culture was pipetted in to each well of a 96 well plate, treated with 1 μL of diluted compound and the plate was incubated at 42°C under microaerobic conditions for 24 h. Growth inhibition was assessed as above.

### Potential for *C. jejuni* acquisition of resistance to the selected inhibitory compounds

Single step and sequential passage resistance assays were performed as described previously with a few modifications (Ling et al., 2015; Xu et al., 2015). The top 12 hit compounds were tested in this experiment. The MIC values for each of these 12 compound as determined in above assay was used for *C. jejuni* resistance studies using lethal (5X MIC) and sub-lethal (0.5X MIC) concentrations.

#### Evaluation of resistance to compounds using sequential passage at sub-lethal dose

Overnight grown *C. jejuni* 81–176 culture was washed and suspended in MH broth to an OD_600_ of 0.05 (~10^7^ CFU). Five milli liter of the OD adjusted fresh culture was mixed with 0.5X MIC of the relevant test compound (concentration allowing > 50% growth inhibition). *Campylobacter jejuni* cultured in 20 μg/mL chloramphenicol, or 50 μg/mL kanamycin, or 2% DMSO, or non-amended MH broth were used as controls. The bacteria were incubated at 42°C with shaking at 200 rpm for 24 h in the dark. Following incubation, cultures were centrifuged at 4700 × g for 10 min at room temperature. The supernatant was discarded; bacteria were resuspended in 5 ml of fresh MH containing 0.5X MIC of the same small molecule. This procedure was repeated 14 times. Following 15 passages, bacterial suspensions were assessed for resistance to the test compound by plating on MH media containing 1X MIC of small molecules.

#### Evaluation of resistance to compounds using lethal dose

*Campylobacter jejuni* grown in MH medium overnight at 42°C was centrifuged at 4700 × g for 10 min at room temperature. The supernatant was discarded and the bacteria were resuspended in MH broth to an OD_600_ of 1.0 (~1 × 10^9^ CFU). Compounds (5X MIC) were mixed with 5 ml of molten MH agar medium and transferred immediately onto small 35 mm petri-plates, agar in the plate was allowed to solidify in the dark. Ten micro liter of culture was transferred to the small molecule containing plates and spread using sterile beads, plates were incubated at 42°C for 5 days in the dark. Bacteria spread on MH agar with 50 μg/mL kanamycin or MH agar alone, were used as positive and negative controls, respectively. After 5 days of incubation, any colonies that developed were assessed for resistance to the test compounds by determining MIC and the MBC as noted above.

### Cytotoxicity of top-12 compounds to human intestinal epithelial cells

We evaluated 12 selected compounds for cytotoxicity to Caco-2 (human colonic carcinoma) cells as described previously (Xu et al., 2015). The Caco-2 cells were obtained from the American Type Culture Collection (ATCC Rockville, MD) and maintained in minimal essential medium (MEM) supplemented with 20% fetal bovine serum (FBS), 1% non-essential amino acid (NEAA; Life Technologies, NY) and with 1 mM sodium pyruvate at 37°C in a humidified 5% CO_2_ incubator. A 96-well tissue culture plate was seeded with approximately 1.4 × 10^5^ cells per well and incubated for 24 h at 37°C in an incubator until a confluent monolayer formed. It was necessary to assess the dose dependent cytotoxicity effect of DMSO as compounds were diluted in DMSO therefore, we determined the cytotoxic effect of varying concentrations of the DMSO vehicle control (1, 2, and 5%) in Caco-2 cells containing 150 μL of media (without FBS) incubated at 24 h. There was no significant difference among the varying DMSO concentrations and their cytotoxicity values. Therefore, we used 1% DMSO in duplicate wells along with other appropriate controls for cytotoxicity experiment.

It is likely that the compounds can bind to serum proteins (FBS) present in the media and thus influence the true cytotoxic effect of the compounds; therefore, we performed the cytotoxicity assay in absence of FBS. The cytotoxicity assay was performed according to manufacturer instructions (Pierce TM; Thermo Scientific, IL) and the percentage of cytotoxicity was calculated by measuring the release of lactate dehydrogenase (LDH) enzyme from the treated cells. Briefly, ~1.4 × 10^5^ cells were grown in a 96-well tissue culture plate containing 150 μL of growth medium. After three washes with medium without supplementation, 1 μL (200 μM) of compound was added to duplicate wells and incubated for 24 h at 37°C in a humidified, 5% CO_2_ incubator. Subsequently, 50 μL of supernatants were collected for measuring LDH release and the degree of cytotoxicity was determined according to the manufacturer instructions.

Three independent experiments were conducted in duplicate samples in each experiment and the average cytotoxicity values were plotted.

### Hemolytic activity of top-12 compounds to sheep RBCs

The hemolytic activity of the top-12 compounds were determined as previously described (Strom et al., 2003; Selin et al., 2015). Briefly, 500 μL of sheep red blood cells (LAMPIRE Biological Laboratories, Pipersville, PA) were washed three times in PBS and resuspended in 5 mL of PBS to prepare a working concentration of 10% RBCs. Two hundred micro liter of the 10% RBCs suspension was incubated with 200 μM concentrations of compounds for 1 h at 37°C in a 96 well plate. After incubation, the plate was centrifuged (4000 rpm, 5 min) and placed on ice for few min. Supernatants from each well was transferred to a fresh 96 well plate and OD of 540 nm was measured. The PBS and 0.1% Triton X-100 were used as negative and positive controls, respectively. A range of 1–10% DMSO was also used as a control. A percent hemolysis was calculated using the following formula:

$$\begin{array}{l} \%\text{ Hemolysis}=(\text{OD of compound}-\text{OD of DMSO/}\\ \text{ OD of Triton-X100)}\times 100 \end{array}$$

Two independent experiments in triplicate were performed and results were expressed as the % average hemolysis.

### Effect of selected compounds on the intracellular survival of *C. jejuni*

To test the effect of selected compounds on *C. jejuni in vitro* clearance, Caco-2 cells were infected with *C. jejuni* 81-176 strain and a 96 well intracellular survival assay was performed as described previously (Malde et al., 2014; Pina-Mimbela et al., 2015). Briefly, a mid-log phase *C. jejuni* 81–176 culture was pelleted by centrifuging at 9500 × g for 10 min and washed three times with Dulbecco Phosphate Buffer Saline (DPBS, Gibco) containing 1% (v/v) FBS and adjusted to the desired OD_600_. Approximately 1.4 × 10^5^ Caco-2 cells/well were seeded in 100 μL of media containing varying concentration of compounds into each well of a 96 well plate and subsequently infected with *C. jejuni* at multiplicity of infection (MOI) 100 in duplicate wells. To determine clearance, Caco-2 cells were incubated with bacteria for 3 h and treated with gentamicin (150 μg/mL) and incubated for additional 2 h. Cells were washed three times with DPBS with no calcium or magnesium and incubated with 1 μL of diluted compounds (200, 100, 50, and 25 μM) for 24 h. Infected cells were washed twice with MEM, lysed with 0.1% (v/v) Triton-X 100. Subsequently, 100 μL of an aliquot from each well was 10-fold serially diluted in MEM and plated on MH agar in duplicate to determine CFUs. The appropriate controls included; (i) infected Caco-2 cell treated with kanamycin or chloramphenicol, (ii) infected Caco-2 cells treated with 1 μL of 100% Trition-X, (iii) infected Caco-2 treated with 1 μL of 100% DMSO. Two independent experiments were carried with duplicate treatments in each experiment and the average CFU value was used to plot the graph.

### Statistical analysis

The *in vitro* clearance ability and cytotoxicity of compounds were analyzed using one-way analysis of variance with mean separation by a least significant difference test at 5% level of significance in GraphPad Prism version 6 software.

## Results

### Primary screen resulted in 781 hit compounds

**Figure 1:**
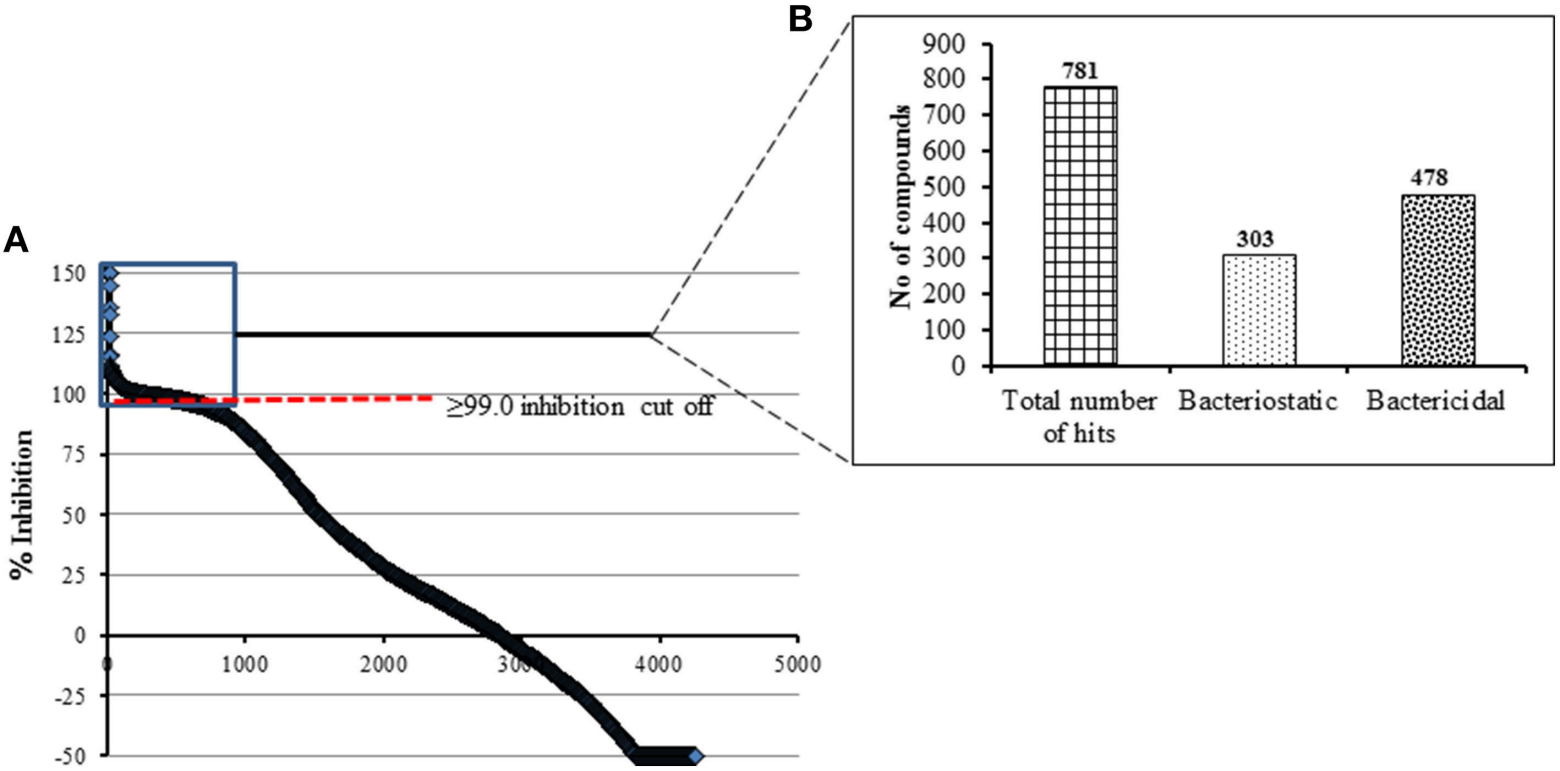
**(A)** Primary HTS of compounds for growth inhibition against *C. jejuni* 81-176 using a pre-selected library of 4182 compounds. A cut off of ≥99.0% growth inhibition resulted in total of 781 hit compounds. These compounds were categorized based on their activity as shown in (**B**).

A total of 4182 small molecules were examined in the primary screening against *C. jejuni* 81-176 for growth inhibition in 96 well plates. During optimization of the screening protocol, we monitored the OD_600_ of each well of the 96 well plates every 4 h for 36 h. We determined that incubation for 24 h yielded maximum number of active hit compounds and that there was no increase in the number of hit compounds upon extended incubation. In similar line, a recent study has also described that the incubation of antimicrobial compounds with different pathogenic bacterial culture between 18 and 24 h is sufficient to read the assay plate in HTS (Mishra et al., 2015). All wells with ≥ 99.0% growth inhibition of *Campylobacter* (compared to controls) were considered a “hit” compound and were further assessed for bacteriostatic and bactericidal properties. We identified 478 bactericidal compounds and 303 bacteriostatic compounds at the end of the primary screening (Figures 1A,B). As the % growth inhibition is directly related with OD of test compound, the compounds that contributed background OD (i.e., inherent ability of certain compounds to absorb OD_600*nm*_) was resulted in negative values for *C. jejuni* growth inhibition (Figure 1A). Overall the observed hit rate in our present study was 18.6% (781/4182) a rate 1.5 times higher than previously reported for the Gram negative *E. coli* BW25113 strain (Wallace et al., 2011).

### Prioritization of bactericidal compounds

From the 478 bactericidal compounds, several common structural motifs were identified. For example 66 compounds contained a piperazine ring as a scaffolding motif. These compounds could be further subdivided into the structural classes shown in Figure S1A, suggesting that common structural features may be found that help to convey anti-campylobacter activity. Similarly, there were 88 compounds containing hydrazone-like functionality (a C = N-N moiety). In many of these compounds, the “hydrazone” was found in pyrazole rings, many of which display very similar substitution patterns. Moreover, there were a few common topological frameworks which seem to appear repeatedly throughout the hit set, sometimes with very slight modifications (Figure S1B).

The purpose of this analysis was to identify compounds which were active against *C. jejuni/coli* and that may possess novel mechanisms of action that convey selectivity for these pathogens, ultimately leading to therapeutically useful antibacterial agents. Based on the relatively high number of bactericidal compounds obtained through the primary screening process, these hits were then filtered using additional criteria to increase the likelihood of success in subsequent drug development efforts. In an attempt to select compounds with physicochemical properties most amenable for drug development, compounds were filtered based on their adherence to: (i) Lipinski's rule of 5 (Lipinski et al., 2001; a measure of the drug-likeness of chemical compounds), (ii) meeting the criteria of the Golden triangle analysis (Johnson et al., 2009), and (iii) lack of obvious reactive functional groups (Bonting et al., 1999). Although nearly all of the compounds in this library met the criteria set forth by Lipinski, active compounds were prioritized based on lower molecular weights and relative LogD values to find compounds that would have better clearance and oral absorption properties. In addition, limiting the MW of compounds to less than 450 encourages later structural optimization of these compounds without making the compound too large. Additional selection criteria were also included to narrow the hitset. These methods included an analysis of structural novelty based on appearances in the chemical literature using SciFinder Scholar searches, diversity of structure based on the primary chemical scaffold, and the ability to rapidly re-functionalize the molecule through application of existing synthetic methods. Using these criteria, 79 unique compounds with bactericidal activity were prioritized for additional screening in order to study these agents in greater detail.

### Forty compounds exhibit a *Campylobacter* broad spectrum effect

**Figure 2:**
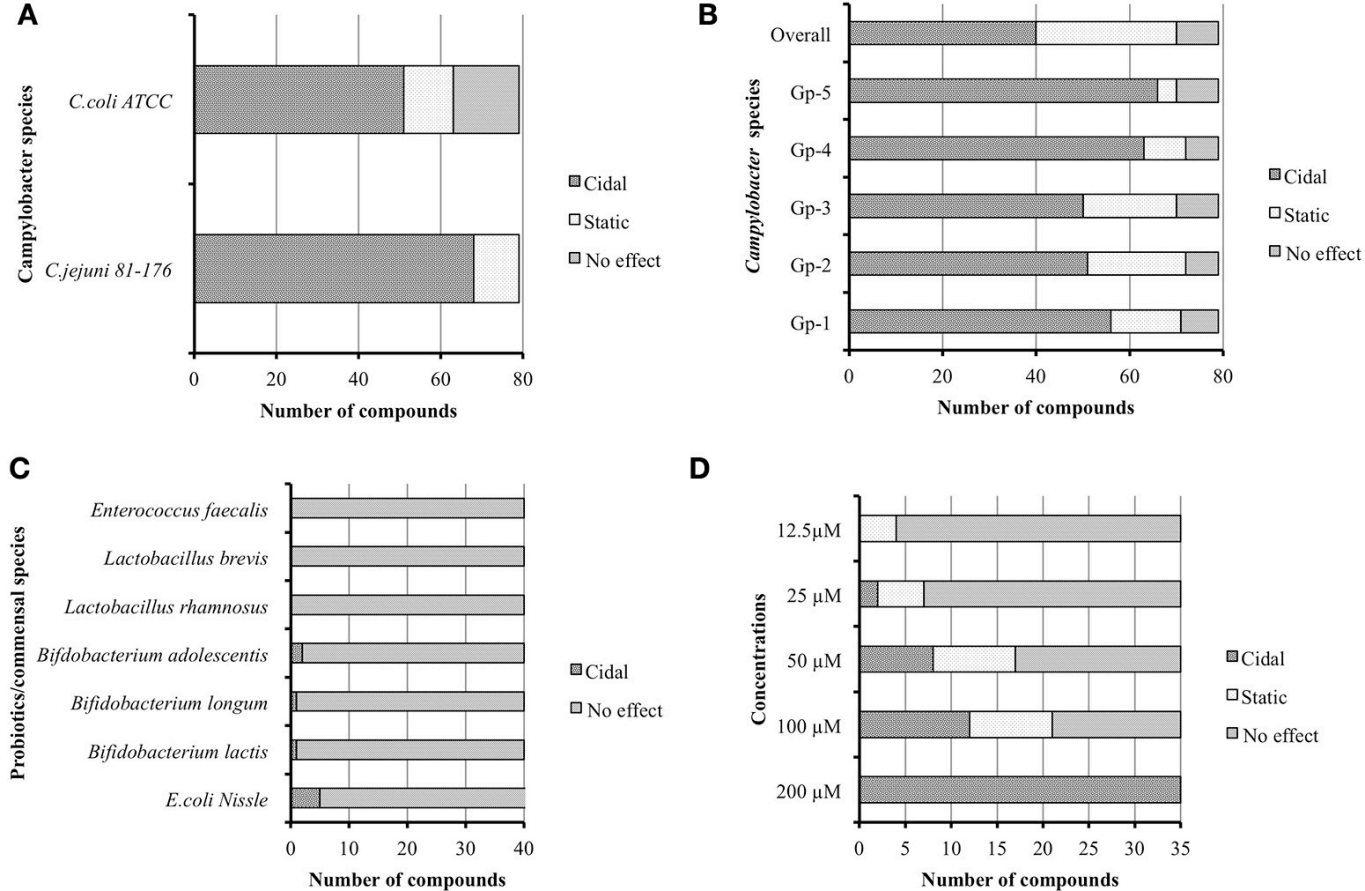
**(A)** Seventy nine re-synthesized hit compounds tested against different *Campylobacter jejuni* and *C. coli.* **(B)** Broad spectrum effect of 79 hit compounds tested against *C. jejuni* isolates from poultry origin grouped as Gp-1 to Gp-5. **(C)** Effect of 40 broad spectrum compounds against probiotics/commensals. **(D)** Minimum inhibitory concentration (MIC) of 35 selected compounds.

Initially we re-confirmed the efficacy of the 79 resynthesized compounds against *C. jejuni* 81–176 and *C. coli* ATCC 33559. The result indicated that 68 and 51 of the compounds were bactericidal, while 11 and 12 were bacteriostatic against *C. jejuni* and *C. coli* respectively, (Figure 2A). Interestingly, 16 out of 79 compounds did not possess any effect on growth of *C. coli* strain.

To test if the prioritized hits possessed similar bactericidal effect against other *C. jejuni* of diverse genotypes, we screened the 79 compounds against poultry isolates of *C. jejuni* (Table S2). The summary of this group screen is described in Figure 2B. In order to determine the overall *Campylobacter* spectrum effect, we considered the small molecules which have bactericidal properties against all groups of *C. jejuni* isolates. We identified 40 such compounds having broad spectrum effect against all 23 tested *C. jejuni* isolates.

### Only five of the 40 broad spectrum compounds affected the growth of commensals/probiotics *in vitro*

Modern antibiotics have an impact on normal gut microflora and are also associated with development of antibiotic resistance (Barbosa and Levy, 2000; Perez-Cobas et al., 2013; Ferrer et al., 2014). Hence we evaluated the 79 compounds for activity against commensal and probiotic bacteria. Among the 40 *Campylobacter* broad spectrum compounds, only five compounds were found to possess bactericidal effect against tested probiotics/commensals. Five compounds inhibited *E. coli* Nissle 1917. Within these five compounds, two compounds also inhibited *B. adolescentis*, while one inhibited *B. longum* and another one inhibited *B. lactis* (Figure 2C). However, no compound showed any effect against other commensals/probiotic strains. Therefore, we omitted the five compounds with some inhibition of commensal/probiotic bacteria and used 35 compounds for further analysis.

### Several compounds possessed MIC as low as 12.5 μM

The minimal concentrations of compounds that inhibit the growth of *C. jejuni* were tested by two-fold serial dilution of each of compound in media as well as in DMSO. The results were reproducible irrespective of diluent used; however, a few compounds precipitated when media was used as a diluent. A recent study also described the in-solubility issues with a few small molecule compounds when dissolved in media for MIC determination (Selin et al., 2015). Hence in subsequent replications, we diluted compounds in DMSO to determine MIC to re-confirm the obtained results (Figure 2D). Of the 35 compounds, 21 compounds had MIC ranging from 100 to 12.5 μM. Among the 21 compounds, 16 were effective at 50 μM, 7 were effective at 25 μM, and 4 were effective at 12.5 μM. We selected top-12 bactericidal compounds with MICs varying from 100 to 12.5 μM concentration for further downstream study.

### Six compounds possessed MBC of ≤ 50 μM against both *C. jejuni* and *C. coli* strains

Minimum inhibitory concentration value not necessarily indicates the killing concentration; therefore, we determined MBC for the top-12 compounds. MBC is defined as the concentration at which 99.9% (below detection limit) of *Campylobacter* organisms are killed. The MBC for each of these compounds were determined as described previously (Ling et al., 2015). The MBC of 8 compounds (Comp 1, 2, 4, 5, 7, 10, 12, and 18) for *C. jejuni* were same as that of their MIC values; while, 4 compounds (Comp 3, 13, 17, and 22) had two-fold higher MBC than the their MIC. Similarly for *C. coli*, 8 compounds (Comp 1, 3, 5, 7, 10, 13, 17, and 22) had MBC value same as that of their MIC value; while, 4 compounds (Comp 2, 4, 12, and 18) had two-fold higher than the their MIC. Overall MIC and MBC values were higher for *C. coli* compared to *C. jejuni* strain. The MIC and MBC for these 12 compounds for both *C. jejuni* and *C. coli* are presented in (Table 1).

**Table 1:** **Summary of classes of top 12 small molecule compounds identified as anti-campylobacter agents**.

		***C. jejuni***		***C. coli***		**Resistance**				***In vitro***
**Class**	**Compound number**	**MIC^a^ (μM)**	**MBC^b^ (μM)**	**MIC (μM)**	**MBC (μM)**	**Single step 5X MIC**	**Passage 0.5X MIC**	**Cytotoxicity (%)**	**Hemolysis (%)**	**Concentration (μM)**
Piperazine	Comp 5	50	50	50	50	No	No	6.5	0	>200
	Comp 2	100	100	50	100	No	No	6.9	0	>200
	Comp 4	50	50	50	100	No	No	4.6	0	>200
	Comp 3	12.5	25	50	50	No	No	11.3	0	>200
Piperidines	Comp 18	25	25	200	>200	No	No	8.8	0	100
	Comp 22	12.5	25	100	100	No	No	12.1	0	100
Aryl amines	Comp 17	12.5	25	50	50	No	No	16.6	0	>200
	Comp 10	50	50	100	100	No	No	27.8	0	50
	Comp 13	12.5	25	50	50	No	No	0	0	>200
	Comp 12	100	100	50	100	No	No	0	0	100
Sulfonamide	Comp 1	50	50	50	50	No	No	30.3	7.4	200
Pyridazinone	Comp 7	12.5	12.5	50	50	No	No	28.0	0	25

a Minimum inhibitory concentration.

b Minimum bactericidal concentration.

### The discovered top 12 small molecules are less likely to induce resistance in *C. jejuni*

Our rationale for performing the resistance studies was to determine the ability of *C. jejuni* strain to develop resistance to these compounds when cultured on solid media with a lethal dose (5X MIC) or in liquid media with a sub-lethal dose (0.5X MIC). The presence of resistant colonies in any of these assays would reduce the attractiveness of the relevant small molecule while an absence of resistant colonies would indicate a likely inability of *C. jejuni* to develop resistance in general. After incubation on solid media amended with a 5X MIC dose of the target compound for 5 days, no resistant *C. jejuni* colonies were observed for any of the 12 compounds tested (Table 1; Figures S2A,B). Following *C. jejuni* incubation at sub-lethal doses (0.5X MIC) in liquid media during 15 passages, identical MICs and MBCs were observed for bacteria that grew at the sub-lethal concentration of small molecules (Table 1). This suggests that *C. jejuni* is less likely to develop resistance to these 12 novel compounds under the tested conditions. Since *C. jejuni* is the major cause of human campylobacteriosis (95% of the cases); (Butzler, 2004) and due to limitation of compounds availability, we only performed resistance studies on *C. jejuni* 81–176. However, for future commercial application of these antimicrobials, more in-depth characterization of potential resistance by other *C. jejuni* strains and *C. coli* is warranted.

### Top 12 compounds had least hemolytic activity but possessed varying cytotoxicity effect

**Figure 3:**
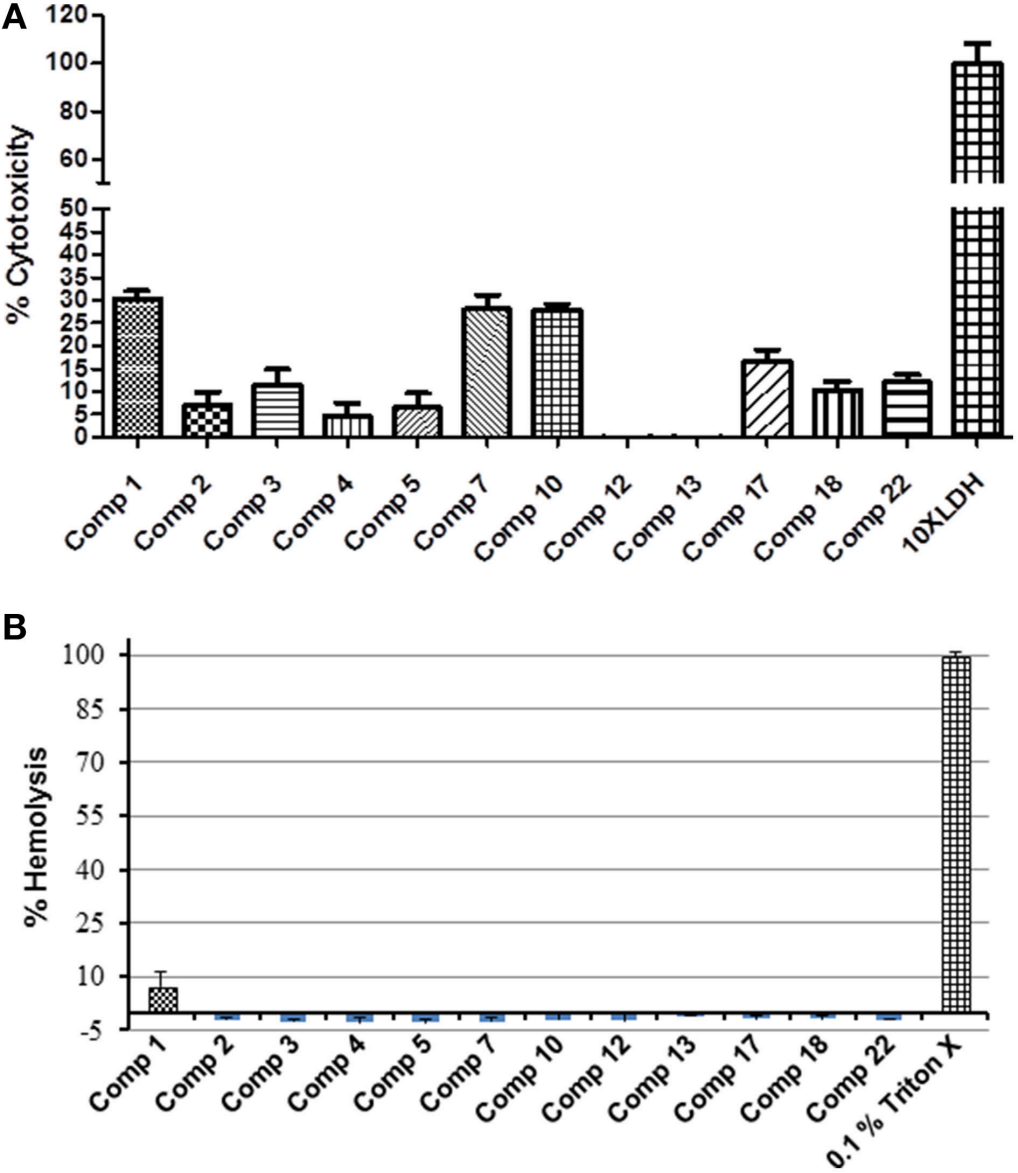
**Percentage cytotoxicity and hemolysis of 12 selected hits**. **(A)** Cytotoxicity was assessed using Caco-2 cells exposed to 1 μL of compound (200 μM) for 24 h. **(B)** The percentage hemolysis was determined by using fresh heparinized sheep blood RBCs incubated with 1 μL of compound (200 μM) for 1 h. All compounds showed significantly less cytotoxicity (*P* ≤ 0.001) compared to positive control.

To avoid the problem of general toxicity to host cells, we determined the cytotoxicity of the 12 prioritized compounds using an established cell culture system (Caco-2 cell line) and sheep RBCs. All of the compounds had a 30% or less cytotoxicity value (Figure 3). Five compounds, (compounds 2, 4, 5, 12, and 13) possessed cytotoxicity value ≤ 10%; of which compound 12 and 13 were least cytotoxic at tested concentration. The cytotoxicity of the compounds was categorized as low, medium, and high (Table S3). Further consistent with cytotoxicity results, except compound 1 (7.4% hemolysis), which also possessed highest cytotoxicity, none of the compounds exhibited hemolytic activity (Table 1).

### All selected 12 compounds are effective *in vitro* clearance of *C. jejuni*

**Figure 4:**
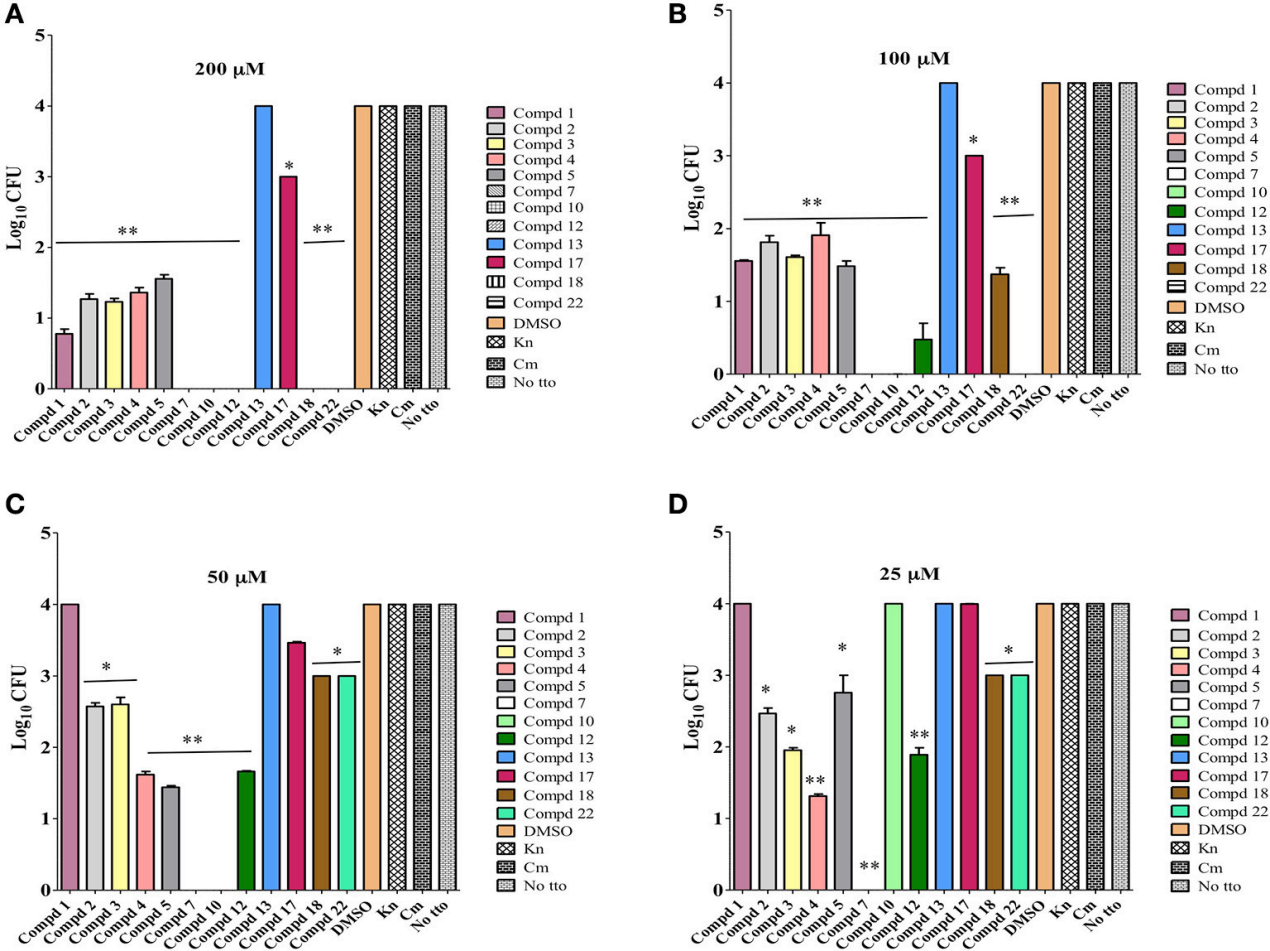
**Intracellular clearance of *C. jejuni* by lead 12 compounds assessed at various concentrations (200, 100, 50, 25 μM) using Caco2 cells infected with highly pathogenic *C. jejuni* 81–176 strain**. Effect of each compound on *C. jejuni* clearance was compared to DMSO treated control. All compounds except compound 13, significantly (^**^ *P* ≤ 0.001, ^*^ *P* ≤ 0.05) cleared intracellular *C. jejuni* at 200 μM **(A)** and 100 μM **(B)** concentrations. Except compound 4, all other compounds significantly cleared intracellular *C. jejuni* at 50 μM **(C)** concentrations. Except compounds 10 and 14, other compounds significantly cleared intracellular *C. jejuni* at 25 μM **(D)** concentrations.

Based on the literature, it is believed that the ability of *C. jejuni* to enter cells of epithelial origin and elicit its cytotoxic effect may reflect an important part of *Campylobacter* pathogenesis in humans (Konkel et al., 1992). Hence Caco-2 cells infected with highly pathogenic *C. jejuni* 81–176 strain were used to determine the intracellular clearance ability of selected lead compounds. With one exception, compound 13, most of the compounds cleared intracellular *C. jejuni* (Figure 4). Even though, compound 13 possessed MIC value of 12.5 μM against *C. jejuni*, it lacked *in vitro* effect. It is possible that this compound is unable to cross cell membrane barrier to induce its effect on *C. jejuni*, as also seen with gentamicin or may be degraded rapidly inside the host cell. At 50 μM concentrations, 9 of the compounds were significantly (*P* ≤ 0.001, *P* ≤ 0.05) effective in reducing 1 log or higher intracellular *Campylobacter* load, however compound 7 and 10 completely reduced the *Campylobacter* load below detection limit (≤10 bacteria/mL). On the other hand at 25 μM concentrations 8 compounds were effective in reducing 1 log or higher intracellular *Campylobacter* load. Surprisingly, compound 7 even at eight-fold dilution (25 μM) did retain its complete intracellular *Campylobacter* clearing ability. This suggests that some of the top-12 selected compounds could be directly exploited as a potential lead compounds for the control of *Campylobacter* in food animals and/or humans. In addition, these compounds and their derivatives can be used to increase the efficacy of conventional antibiotics for which *C. jejuni* is resistant to as demonstrated recently by use of phenolic compounds (Oh and Jeon, 2015).

### Structural analysis of the top 12 lead compounds

**Figure 5:**
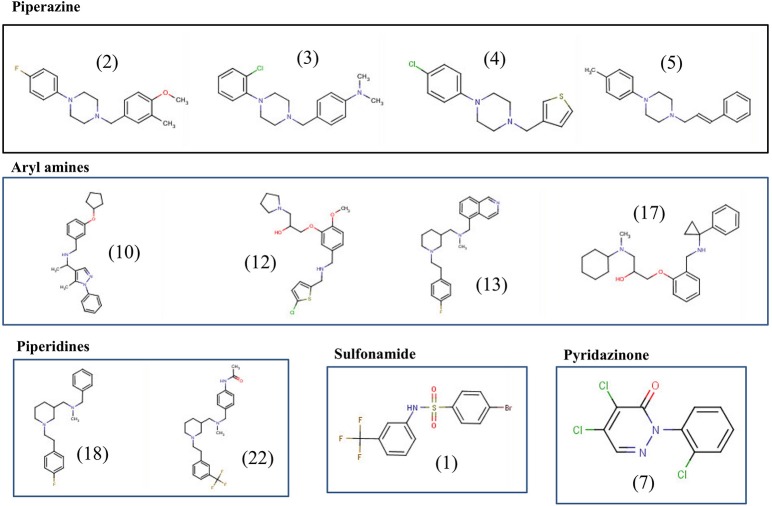
**Chemical structures of the top 12 potent small molecules inhibitory to *C. jejuni* identified in this study**.

The final 12 identified compounds belonged to five chemical classes; piperazines, piperidines, aryl amines, sulfonamide, and pyridazinone (Figure 5). The compounds in the piperazine class displayed lower cytotoxicity and lower MIC values compared to those in the aryl amines class. Although the compounds in the piperidines class had lower MICs values compared to those in the piperazine class, they had little higher cytotoxicity to Caco-2 cells. Overall, the compounds in the sulfonamide and pyridazinone classes displayed significantly higher cytotoxicity value compared to the other three classes. A comprehensive summary of the chemical class of the compounds with their MIC, MBC, resistance data, cytotoxicity and hemolysis, and *in vitro* clearance ability are provided in Table 1.

## Discussion

**Figure 6:**
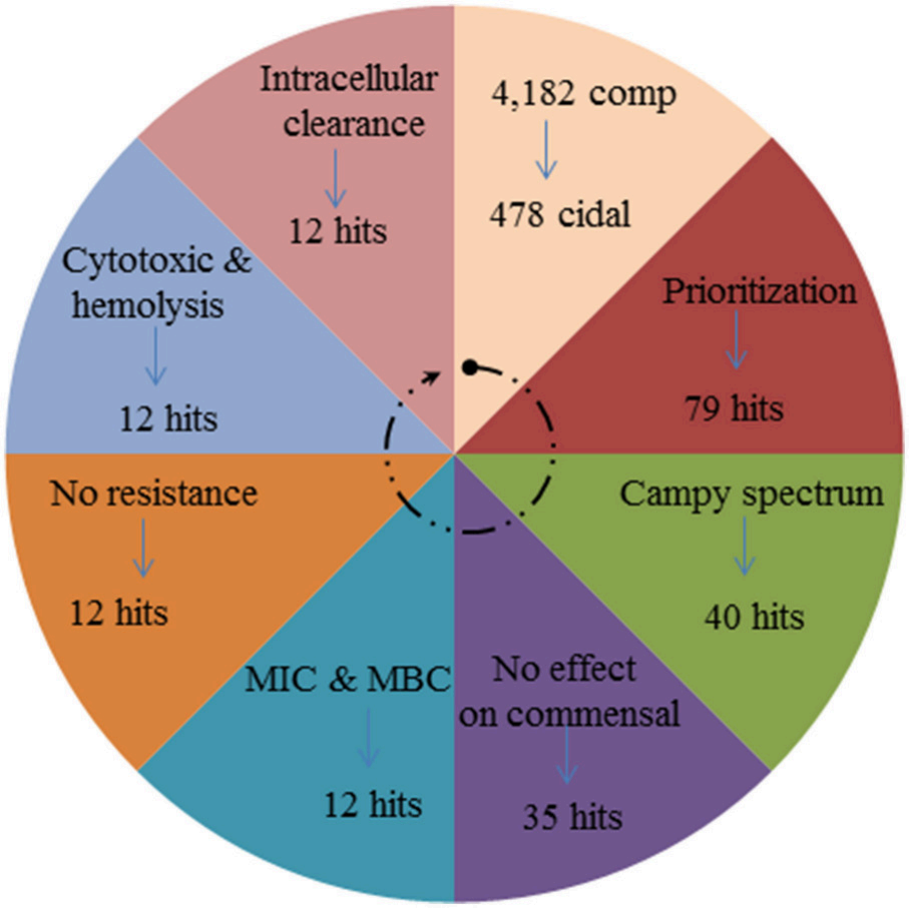
**Wheel diagram showing the summary of number of compounds filtered at each step of primary and secondary screening to discover top-12 anti-Campylobacter small molecule compounds**.

In the current study, we report on the results of an HTS-growth inhibition screen to identify novel anti-campylobacter compounds. We identified 12 novel small molecules with a suitable range of characteristics (see Figure 6). The initial goal of our project was to exploit the pre-selected library to identify hits that completely inhibit the growth of *Campylobacter.* The HTS screening platform has proven useful in discovery of small molecule candidates that inhibit bacterial growth in whole cell based or target specific assays (Hong-Geller, 2013). Previously in a similar study, it was found that small molecules for antimicrobial activity can be discovered by assessing % of growth inhibition in a 96 well format in *E. coli* and *P. aeruginosa* (De La Fuente et al., 2006) It was found that HTS method is robust to screen small molecules and it relies on the classical correlation between bacterial growth and turbidity (De La Fuente et al., 2006). The compounds producing > 60% of turbidity inhibition was selected as a hit in primary screening and was re-verified at tested concentration for growth inhibition. In the prior study (De La Fuente et al., 2006), which used compounds at 12.5 μM, the hit rate was 0.024% for *E. coli* and 0.005% for *P. aeruginosa*. However, in the current study, the hit rate by assaying complete *Campylobacter* growth inhibition at 12.5 μM was 0.096% of the 79 selected compounds which was approximately four-fold higher than reported previously for *E. coli* (Butzler, 2004). In the current and the prior studies (De La Fuente et al., 2006; Wallace et al., 2011), the cell-based HTS completely depends on bacterial growth inhibition and hence, may not be able to select a hit that target virulence genes without influencing the growth. It is likely that the most of the antibiotics with bactericidal activity and that lack ability to induce resistance have non-specific mode of action (Ling et al., 2015). However, in the present study, the discovered top 12 compounds were bactericidal to *Campylobacter* isolates and no resistance development was observed (Figure 2; Table 1). In a recent study it was shown that a newly discovered soil origin antibiotic, though showing bactericidal action against *Staphylococcus aureus*, did not induce resistance and that the mode of action of the novel antibiotic included specific inhibition of peptidoglycan biosynthesis (Ling et al., 2015). Further studies are needed to understand the mode of action for the 12 anti-*Campylobacter* compounds discovered in the current study.

Much of the focus for discovery of novel therapeutic agents is centered on the need for agents that have a shorter treatment length with no obvious side effects and which are unlikely to induce resistance in the pathogen. The piperazine class of compounds meet these critical characters (Patel and Park, 2013) and were amongst the anti-*Campylobacter* compounds found in the current study. Piperazine derivatives have been extensively associated with numerous biological functions like antibacterial, anti-mycobacterial, antifungal, anticancer, anti-HIV, antipsychotic, anticonvulsant, antimalarial, and antioxidant (Patel and Park, 2013). Further it was observed that varying substitution of free nitrogen atom of piperazine ring leads to enhanced biological significance of the generated molecules (Patel and Park, 2013). Substituents may be either aliphatic, aromatic or heteroaromatic compounds. For examples, piperazine rings bearing thiazoloquinolines and thiazolocoumarins compounds have been shown to be significantly active against Gram-positive (*S. aureus* and *Bacillus cereus*), Gram-negative (*E. coli, P. aeruginosa* and *Klebsiella pneumoniae*) and fungal species (*Aspergillus niger* and *Candida albicans*) at MICs between 12.5 and 50 μg/mL (Patel and Park, 2013). Therefore, in our future studies, we will generate synthetic derivatives of key compounds by substituting side chains with aliphatic or aromatic compounds and test their efficacy against *C. jejuni* and other relevant Gram negative food borne pathogens.

In the current study, we also identified other four chemical classes of compounds that have varying potency (MICs) and toxic effects. For example, the piperidine and aryl amine chemical classes showed a relatively higher toxic effect compared to the piperazine derivatives. However, the piperidine class compounds had lower MICs value than the other two classes. Sulfonamide and pyridazinone classes had both lower MICs and but possessed higher cytotoxicity values. Surprisingly, the pyridazinone class derivatives had MIC ≤ 12.5 μM. Further, this class has exhibited an ability to clear *C. jejuni in vitro* at all the tested concentrations (≤25 μM). Like the piperazine derivatives, the pyridazinone derivatives also showed a wide spectrum of biological activities (Ibrahim et al., 2013). In summary, we have identified 12 potential lead compounds (see Figures 5, 6) that are active against *C. jejuni* and future work on; increasing the efficiency of the compounds by downstream modification, target identification, and biologically active functional groups identification, must be explored.

## Author contributions

Conceived and designed the experiments: AK, MD, XX, and GR. Performed the experiments: AK, MD, RP, XX, and YH. Analyzed the data: AK, JA, JF, CN, JT, PB, and GR. Wrote the paper: AK, PB, CN, and GR.

## Funding

This research was conducted within the Poultry CRC, established and supported under the Australian Government's Cooperative Research Centres Program. Dr. Rajashekara's laboratory is supported by the funds from Ohio Agricultural Research and Development Center (OARDC), The Ohio State University, and the Agriculture and Food Research Initiative (AFRI) grant# 2012-68003-19679, U. S. Department of Agriculture. The funders had no role in study design, data collection and analysis, or preparation of the manuscript.

### Conflict of interest statement

The authors declare that the research was conducted in the absence of any commercial or financial relationships that could be construed as a potential conflict of interest.

## Supplementary material

Figure S1 **(A)** An example of a common structural motif found in the *Campylobacter* (bactericidal) hit set. (**B**) A topogical framework repeated in several hits.

Figure S2 **Evaluation of ability of *C. jejuni* to develop resistance to selected top-12 compounds:** (**A**) Single step spontaneous resistance study, where “No culture” refers to MH plate without *Campylobacter* culture. (**B**) Sequential passage study, where “DMSO” refers to MH plate containing 1% DMSO + *Campylobacter* culture. In both study “negative hit” refers to X compound that did not inhibit the growth of *Campylobacter*. Assays were performed using two replicates.

Table S1 **List of bacterial strains, media and culture conditions used in the study**.

Table S2 **Classification of poultry *C*. *jejuni* isolates based on their prevalence and SNP type**.

Table S3 **Classification of 12 lead compounds based on their cytotoxicity**.
