# Supplementary figures and images for: Novel Anti-Campylobacter Compounds Identified Using High Throughput Screening of a Pre-selected Enriched Small Molecules Library
Source: Front Microbiol. 2016 Apr 6;7:405. doi: 10.3389/fmicb.2016.00405 (PMC4821856; doi:10.3389/fmicb.2016.00405)

Fig. S1 A and B

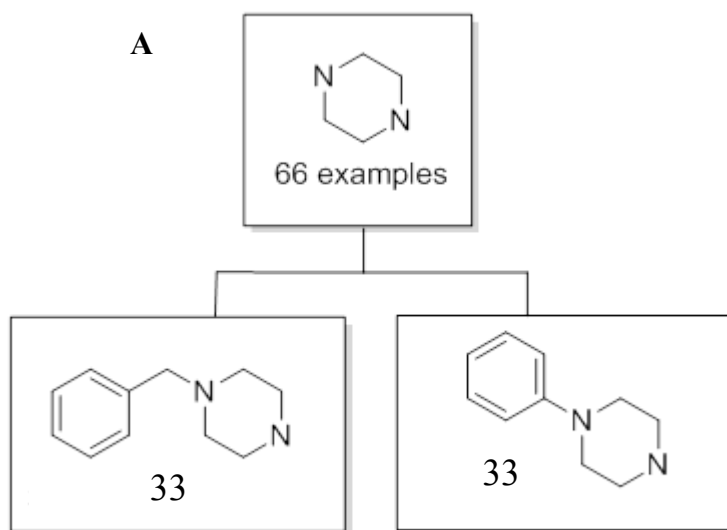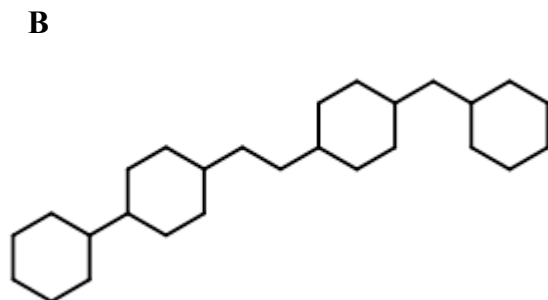

Fig. S2 A and B

**A**

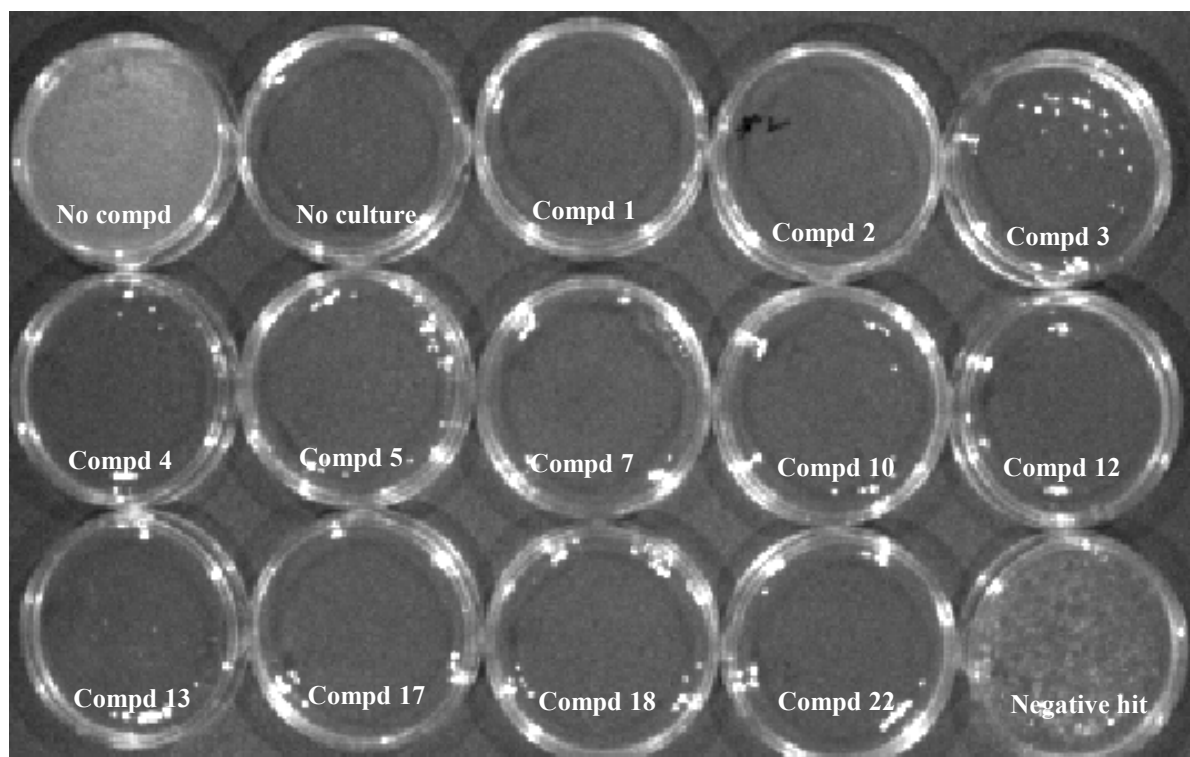

**B**

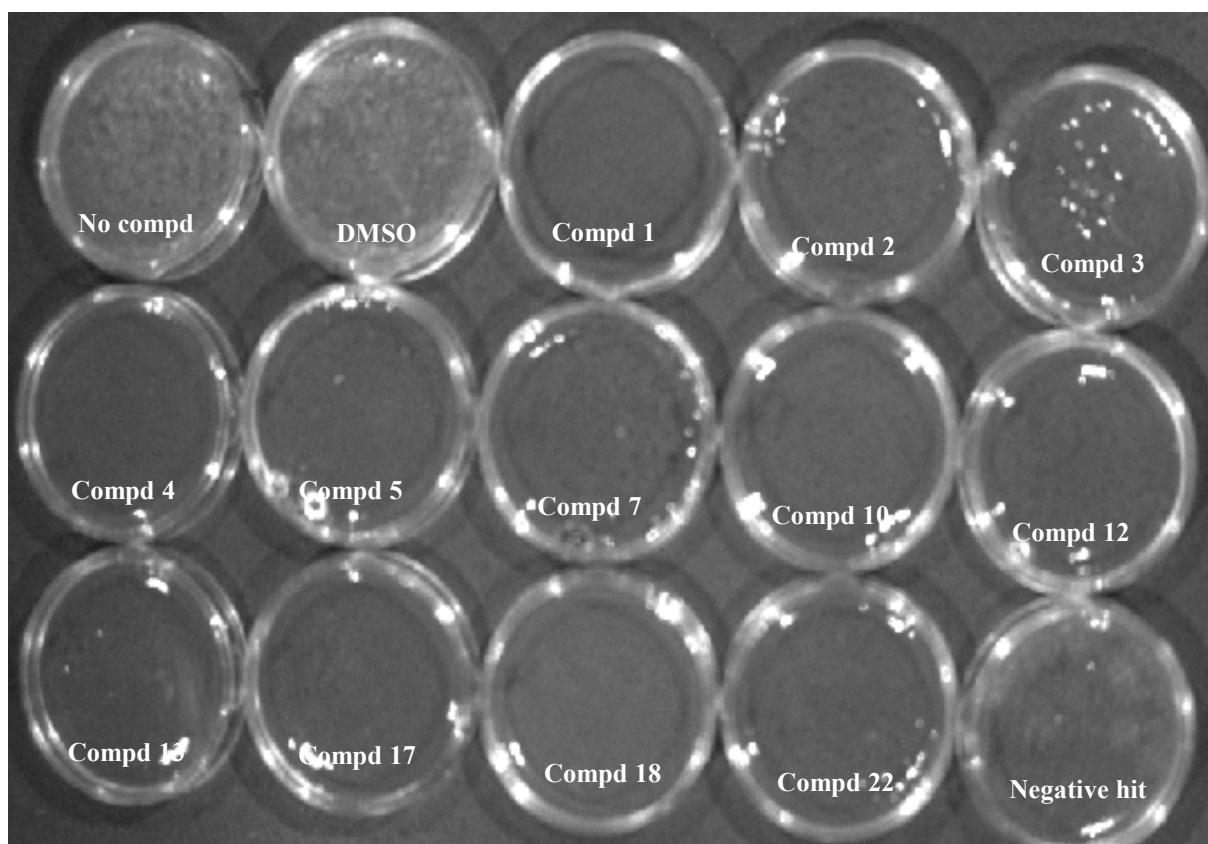

Supplement: Figure S1 — (A) An example of a common structural motif found in the Campylobacter (bactericidal) hit set. (B) A topogical framework repeated in several hits. [file Image1.PDF]
