# Supplementary material for: Novel Anti-Campylobacter Compounds Identified Using High Throughput Screening of a Pre-selected Enriched Small Molecules Library
Source: Front Microbiol. 2016 Apr 6;7:405. doi: 10.3389/fmicb.2016.00405 (PMC4821856; doi:10.3389/fmicb.2016.00405)
Supplement: Table S1 — List of bacterial strains, media and culture conditions used in the study. [file Table1.PDF]

Table S1: List of bacterial strains, media and culture conditions used in the study

Bacterial spp.	Strain	Media	Culture conditions	Reference/Source
Campylobacter jejuni	81-176	Mueller Hilton (MH) agar/broth	42˚C, microaerobic	Dr. Qijing Zhang
Campylobacter coli	ATCC 33559	Mueller Hilton (MH) agar/broth	42˚C, microaerobic	Dr. Qijing Zhang
Campylobacter jejuni	AU13, AU17, AU20, AU41, AU33, AU50, AU12, AU16, AU38, AU22, AU47, AU19, AU31, AU18, AU45, AU46, AU35, AU44, AU49, AU24, AU28, AU21, AU39	Mueller Hilton (MH) agar/broth	42˚C, microaerobic	Jillian Templeton field isolates
Enterococcus fecalis		Man Rogosa Sharpes (MRS) broth	37˚C, anaerobic	Lab collection
Lactobacillus brevis		Man Rogosa Sharpes (MRS) broth	37˚C, anaerobic	Lab collection
Lactobacillus rhamnosus	LGG- ATCC 53103	Man Rogosa Sharpes (MRS) broth	37˚C, anaerobic	ATCC, Manassas, VA, USA
Bifidobacterium adolescentis		Man Rogosa Sharpes (MRS) broth with 0.5% Cysteine	37˚C, anaerobic	Lab collection
Bifidobacterium longum		Man Rogosa Sharpes (MRS) broth with 0.5% Cysteine	37˚C, anaerobic	Lab collection
Bifidobacteriun lactis	Bb-12	Man Rogosa Sharpes (MRS) broth with 0.5% Cysteine	37˚C, anaerobic	Christian Hansen Ltd., Hørsholm, Denmark
Escherichia coli	Nissle 1917	Luria-Bertani (LB) broth	37˚C, aerobic and 200 rpm	Dr. Ulrich Sonnenborn, Ardeypharm GmbH, Herdecke, Germany

Table S2: Classification of poultry *C. jejuni* isolates based on their prevalence and SNP type [23]

Groups	*C. jejuni* genotypes	SNP Type
Gp-1	Au-13	SNP-1
	Au-17	SNP-1
	Au-20	SNP-5
	Au-41	SNP-5
Gp-2	Au-33	SNP-6
	Au-50	SNP-6
	Au-21	SNP-36
	Au-39	SNP-36
Gp-3	Au-12	SNP-11
	Au-16	SNP-11
	Au-38	SNP-11
	Au-22	SNP-12
	Au-47	SNP-12
Gp-4	Au-19	SNP-14
	Au-32	SNP-14
	Au-18	SNP-15
	Au-45	SNP-15
	Au-46	SNP-15
Gp-5	Au-35	SNP-20
	Au-44	SNP-20
	Au-49	SNP-20
	Au-24	SNP-22
	Au-28	SNP-22

Table S3: Classification of 12 lead compounds based on their cytotoxicity.

**Categories**	**% cytotoxicity**	**Number of compounds**	**MIC range µM for *C. jejuni***
Less	0 to ≤5	3	50-12.5
Medium	6 to 10	3	100-50
High	11 to 30	6	100-12.5
